# Designing a Co-creation System for the Development of Work-process-related Learning Material in Manufacturing

**DOI:** 10.1007/s10606-021-09420-5

**Published:** 2022-01-12

**Authors:** Tim Weinert, Matthias Billert, Marian Thiel de Gafenco, Andreas Janson, Jan Marco Leimeister

**Affiliations:** 1 Information Systems, University of Kassel, Pfannkuchstrasse 1, 34121 Kassel, Germany; 2 Economic and Business Education - Professional Teaching and Learning, University of Kassel, Henschelstrasse 2, 34127 Kassel, Germany; 3 Institute of Information Management (IWI-HSG), Müller-Friedberg-Strasse 8, 9000 St. Gallen, Switzerland

**Keywords:** Action-design research, Co-creation, Cognitive load, Manufacturing, Situated learning material, Vocational education and training

## Abstract

The increasing digitalization and automatization in the manufacturing industry as well as the need to learn on the job has reinforced the need for much more granular learning, which has not yet impacted the design of learning materials. In this regard, granular learning concepts require situated learning materials to support self-directed learning in the workplace in a targeted manner. Co-creation approaches offer promising opportunities to support employees in the independent design of such situated learning materials. Using an action-design research (ADR) approach, we derived requirements from co-creation concepts and practice by conducting focus group workshops in manufacturing and vocational training schools to develop design principles for a co-creation system that supports employees through the creation process of work-process-related learning material. Consequently, we formulate four design principles for the design of a collaborative learning and qualification system for manufacturing. Using an innovative mixed methods approach, we validate these design principles and design features to demonstrate the success of the developed artifact. The results provide insights regarding the design of a co-creation system to support learners in the co-creation of learning material with the consideration of cognitive load (CL). Our study contributes to research and practice by proposing novel design principles for supporting employees in peer creation processes. Furthermore, our study reveals how co-creation systems can support the collaborative development of learning materials in the work process.

## Introduction

Due to the general digitalization and automatization of the manufacturing industry, there has been an increase in the complexity of industrial manufacturing processes (Fuller et al. 2020). This increase has had a strong impact on the necessary skills and knowledge of employees who have to deal with changing work conditions every day (World Economic Forum 2018). Simultaneously, it is assumed that almost 60 % of the work activities in the production sector are potentially automatable in the future, which solidifies the need for structured education and further training of blue-collar workers (Ellingrud et al. 2020). These training processes often draw from established instructional design models, like cognitive apprenticeship (Brown et al. 1989), or more contemporary approaches of social workplace learning (Erpenbeck et al. 2016), like collaborative virtual learning environments (Lu et al. 2018). The models are often termed “on-the-job training” and are used to deal with the decreasing half-life of skills acquired at the workplace (Senderek 2016). A thorough integration of learning in the work process can be indicated as a commonality of most of these concepts, which certainly contributes to their success (Dehnbostel 2008). Furthermore, manufacturing facilities as learning environments are associated with several challenges for employees, such as high noise levels and the constant monitoring of production processes, which complicates the establishment of action-oriented learning processes (Senderek 2016) and results in a cognitive overload during knowledge acquisition. In addition, commonly used learning material may not support the usage of such concepts and learning processes in manufacturing in a significant manner.

Further, learning material used for in-company training in the manufacturing industry rarely meets the requirements of the characteristics of the individual workplace. This is because learning materials are created by employees who are not directly familiar with everyday practical work processes. Consequently, the specifications of the work processes are not closely portrayed in the design of the learning material - for example, with process workarounds - and, thus, lack the didactical foundation to be used for learning in an effective manner (Dehnbostel 2008). In addition, the examination of existing documentation, through a previously conducted work-process analysis (Spöttl 2007), revealed outdated descriptions of manufacturing processes and unstructured problem-solving documentation. If there are images in the documentation, the affiliation of the text to the action instruction is not immediately obvious to the employees. This analysis substantiated our impression of the inadequacy of these documents for in-company training in the manufacturing industry. Different knowledge and expertise approaches attempt to meet these challenges by creating means to document knowledge in the process of working (Carvalho et al. 2018). In this regard, typical approaches face the problem that the knowledge required for production is highly contextual and, therefore, cannot be readily manifested (Carvalho et al. 2018; Nakano et al. 2013). To overcome these challenges, co-creation concepts can be integrated to support the creation of work-process-related learning material (Senderek 2016). Furthermore, co-creation or peer creation concepts describe the development of learning material by employees, for employees (Auvinen 2009). There is a huge variation in the types of co-creation concepts applied worldwide that focus on different cases, actors, and targets (Bovill 2020) - for example, to provoke knowledge transfer in collaborative processes in organizations (Oeste-Reiss et al. 2016; Bittner et al. 2021), to enhance the learning success of students in higher education (Wegener and Leimeister 2012a; Coetzee et al. 2015; Janson et al. 2016), or to involve students in teaching and designing academic development courses (Howson and Weller 2016). Simultaneously, the creation of content has positive effects on the diverse employees involved in the co-creation process - for example, increasing autonomy, self-regulation, and responsibility (Deeley and Bovill 2017); improving performance (Bovill 2014; Coetzee et al. 2015); or improving critical reflection and communication skills (Deeley 2014). Thus, co-creation enables the design of situated adapted learning material by employees, which, in turn, enables the adaptation of the learning material to the work process (Pletz and Zinn 2020). This situated and work-process-related learning material has decisive advantages over abstracted and generalized learning material, as it is easier for employees to understand and is just-in-time available, thereby counteracting cognitive overload (Lave and Wenger 2011; Brown et al. 1989).

However, co-creation processes in manufacturing pose specific challenges for employees, as, first, they do not have the didactic competence to create learning material (Oeste-Reiss et al. 2016); second, co-creation processes that take place in the work process create a rather hostile environment for learning (Erpenbeck et al. 2016; Hackman and Oldham 1975), thereby placing an additional CL on employees (Pletz and Zinn 2020). To meet these challenges, it is necessary to design the co-creation process in such a manner that employees are systematically guided and instructed through the creation to support them in taking into account at least rudimentary didactical aspects in a manner that enables the creation of high-quality work-process-related learning material (Weinert and Thiel de Gafenco 2020). This work-process-related learning material, which is developed through the co-creation process, is also termed “micro-learnings” (Schmidt 2007). Attempts to address the outlined shortcomings may fail to consider the detrimental effects of the actual work environment in engaging with knowledge and co-creation systems (Choi et al. 2014). In particular, the effects on cognitive performance in the co-creation process are not yet sufficiently investigated (Caskurlu et al. 2020).

For both practical and scientific research purposes, designing a co-creation system following recent insights from educational psychology in general and the cognitive load theory (Choi et al. 2014), situated learning theory (Lave and Wenger 2011), and instructional design (Janssen and Kirschner 2020) are promising for the purpose of overcoming these challenges, thereby enabling companies to exploit the benefits of the co-creation of learning material. Against this background, our work pursues the goal of developing a co-creation system that supports blue-collar workers in the development of work-process-related learning materials on the shop floor in manufacturing. Hence, the research aim is formulated in the following manner:

- **
RQ: How should a co-creation system be designed to support employees in the co- creation of work-process-related learning material under the consideration of cognitive load?**

We contribute to the computer supported cooperative work (CSCW) community by investigating how a co-creation system in manufacturing must be designed to be advantageous in terms of employee-driven knowledge development and documentation. Furthermore, we show that the co-creation process has positive effects on the learning outcomes of employees in on-the-job training. In our results, we show validated design principles for the design of co-creation systems that support blue-collar workers in the development of work-process-related learning materials. Moreover, we show that by systematically considering the cognitive load, the effort required to develop the learning materials in the work process can be reduced so that the development can be better integrated into the work process.

Furthermore, to address the research aim, ensure the development of the necessary practical background of the study, and be able to systematically consider the requirements of the blue-collar workers, we follow an action-design research (ADR) approach (Sein et al. 2011). For this purpose, in Section 1, we explained the problem and the research context. In Section 2, we explain the theoretical background of how a rigorous instructional design can support blue-collar workers in the co-creation process of learning material. In Section 3, we explain the research method in more detail and the design process of the co-creation system. Following the approach of Sein et al. (2011), we add a short discussion of the most important findings after each building, intervention, and evaluation (BIE) cycle, which leads to a general discussion in Section 4. In the final section, we outline the contribution of our work as well as its limitations.

## The problem and the research context

Following the approach of Sein et al. (2011), we present the underlying problem of the study from a practical perspective. In order to do this, we declare the problem from the viewpoint of our partner company and further show that the problem is not only an individual case but a deeper problem in the dissemination of knowledge in the manufacturing industry. The study at hand is further embedded in a larger, multi-year-project (see Thiel de Gafenco et al. (2018) for further details concerning the project). Based on the most important theoretical concepts and related work from the perspective of the CSCW community, we relate the concepts to the identified problem.

### Description of the problem

The idea of the project started with the operation managers of a manufacturing plant of an international health care company, who compared the documented process sequences with the real-work sequences of blue-collar workers in manufacturing during the process of revision of the material. They quickly realized that many actual work processes did not correspond to the documented work processes. Simultaneously, it became evident that the documentation of instructions for solving problem cases had been conducted adequately but in a manner that other employees could not handle. It is a widespread problem with which those responsible in the company have to struggle - knowledge is not prepared in a structured manner so that other people can benefit from it (Zhao et al. 2018; Sensuse et al. 2018). While the documentation process may be perceived as too complex and, therefore, terminated early, workshop employees develop, share, and use tacit knowledge in the performance of their daily tasks. However, the process of knowledge-sharing, particularly on the shop floor, still proves to be challenging for employees (Nakano et al. 2013) - for example, due to a lack of communication skills (Miyake and Nakano 2007). Moreover, it can be noted that articulating knowledge outside the immediate context of work is considered difficult for many individuals (He and Wei 2009; Carvalho et al. 2018).

The managers of the manufacturing plant reacted to these complaints in a time-honored manner: They marked the deviations and attempted to record the new work processes in the documentation or provided instructions to follow the recorded procedures. The operation managers did not investigate why the employees do not use the documentation in case they encounter a problem. However, employees are often trained on the job rather and do not use the documentation material. An employee, who is part of the vocational education and training (VET) expert team of the underlying research project of this article, was contacted by the managers; they were able to confirm that this is a general trend, particularly in manufacturing-related work environments. The existing documentation and learning material rarely met the requirements of the blue-collar workers, particularly regarding their usage as a memory aid in the work process as well as to meet their requirements for work-process-related learning material.

To gain a deeper understanding of the problem, a project team was formed that comprised employees of the company, a participating vocational school, and researchers from a local university. The project team studied the existing literature on VET practices in manufacturing to verify the problem and found that these knowledge transfer problems were rather common. Amongst several reasons, those indicated were errors in the design of the learning material as well as regarding their applicability to real-life situations (Senderek 2016). Our findings appeared to support our assumption that the main reason for the poor quality of the learning material was related to the lack of support and experience of the employees in the creation process of these materials as well as the lack of involvement of the blue-collar employees from the actual work processes and less to the ability of the employees. Thus, approaches are required to overcome the described deficiencies but also leverage the potential of blue-collar employees for obtaining better learning materials.

### Co-creation of learning material for knowledge-sharing in manufacturing

In different contexts, there is an increasing interest in research and practice regarding co-created learning and teaching - for example, in higher education (Bovill 2020) or VET (Weinert and Thiel de Gafenco 2020). The terms peer creation, students as partners, partnership, and co-creation are often used interchangeably (Bovill 2020; Wegener and Leimeister 2012a). We refer to the term “co-creation” because the term “partners” often implies a level of equality among actors in the co-creation process (Cook-Sather 2018). Generally, the concept of co-creation is based on theories of social constructivism and refers to learning with and from colleagues (Wegener and Leimeister 2012a). The development of learning material that is created by and for learners is a prominent approach to helping people in their learning process (Auvinen 2009). Hence, co-creation concepts comprise mechanisms with insights into how people can develop artifacts, like learning material, in the learning process. The peers add value to the material by making their knowledge available in the form of learning material (Oeste-Reiss et al. 2016). This material contains three defining aspects: (1) It is published in some way on a platform, (2) the creation of the material demands a certain amount of creative effort and (3) the material is created outside normal routines (OECD 2007). Therefore, the creation process is often not commercially controlled but is triggered intrinsically. Furthermore, the developed learning material is mostly situational, consisting of short individual contributions, discussions, or comments (Wegener and Leimeister 2012b). However, Bovill et al. (2016) indicate that a concrete co-creation process must be adapted to the user’s context in order for it to be successful. In particular, in the manufacturing industry, where numerous work processes have to be documented, it is possible that at least a few aspects of the co-creation process can be controlled extrinsically (e.g., through money) rather than intrinsically (e.g., by self-motivation). Although research has empirically demonstrated successful participant inclusion and the resulting positive effects in a variety of different scenarios (Deeley 2014; Wegener and Leimeister 2012b; Martin and Bolliger 2018), the inclusion of participants in the creation process remains a central challenge in the co-creation process (Bovill et al. 2016). Against the background of the different occupational fields and levels of competence of employees in the manufacturing industry, it is necessary to investigate how co-creation processes that take into account the special characteristics of manufacturing can be designed.

### Situated work-process-related learning material

The situational character of the created learning material in the co-creation process is highlighted by a few authors as problematic (Emerson and Berge 2018; Wegener and Leimeister 2012b) because although co-creation processes have several positive effects on involved learners (i.e., Bovill 2020), developed learning materials often cannot be used as modular and independent learning units. However, the situational character of the learning material can also have positive effects on learning (Lave and Wenger 2011). Against this background, the theory of situational learning assumes that the acquisition of knowledge is always related to the situation that is part of an activity, context, and culture in which the knowledge is *developed* and *used* (Brown et al. 1989). In terms of the co-creation process itself, this implies that the learning material must be *developed* in the context in which it must be used subsequently (Pletz and Zinn 2020). Therefore, learning (*used*) is effective when a situational connection to the professional and working world is established (Lave and Wenger 2011; Pletz and Zinn 2020; Young 1993). By referring to actual situations in the learning material, it is easier for learners to understand the background and this reduces the CL that occurs when acquiring new knowledge (Ayres 2020). By referring to a real, known context in the learning material, split-attention effects can be avoided, as learners would otherwise have to bring together information from two different contexts (Tabbers 2002). Based on these ideas, several concepts were developed, which are collectively known under the term “Communities of Practice” (Su et al. 2012). First discussed by Lave and Wenger (1991), the term underwent several transformations. Now encompassing their function as a conceptual lens of situated social construction of meaning, concrete communities focused on knowledge-sharing within an organization (Cox 2005). It may be necessary that practitioners of CSCW be aware of these ambiguities and embrace its diversity (Su et al. 2012).

Apart from the positive influence on the subsequent user of the situational learning material, the co-creation process within the working process may also have positive effects on the performance of the learners as creators themselves (Korthagen 2010). The understanding of learning as a situated experience - that is, an experience resulting from involvement and co-participation in a particular context as well as with other colleagues who generate and use that learning, underpins the socio-constructivist approach to VET advocated by certain scholars (La Cámara de Fuente and Comas-Quinn 2016; Lave and Wenger 2011). From the constructivist approach, learners generate knowledge by working with real-world problems (Gupta and Bostrom 2009). Related to this, the co-creation of work-process-related learning material can support the learning process by integrating the creation process into the real-work situation of the individual learners to create the necessary situational reference for the learning material.

Against this background, we assume that the situated co-creation of work-process-related learning material has a positive influence on the learning performance of the actors - that is, the learners involved.

### The nature of cognitive load and instructional design in collaborative work

Even though the situational property of learning material has a positive influence on the learners involved in the creation, knowledge-sharing has always been a major challenge for employees in manufacturing (Ackerman et al. 2013; Lewkowicz and Liron 2019). While technologies can help employees to create knowledge in a structured manner in the co-creation process (Hoffmann et al. 2019), the cognitive capacities of people often limit their ability to share knowledge in practice. Furthermore, it can be stated that this limitation of cognitive abilities has not been sufficiently taken into account in these practice-oriented works (Hoffmann et al. 2019). Furthermore, the cognitive load theory (CLT) - as a part of instructional design models - considers “working memory constraints as determinants of instructional design effectiveness” (Sweller et al. 1998, p. 251). The working memory, also called short-term memory, is responsible for organizing and manipulating information into new or existing schemas and encoding them to store in long-term memory (Caskurlu et al. 2020; Paas et al. 2003). In contrast to long-term memory, short-term memory has a limited capacity and it can only hold and process a limited amount of information simultaneously (Paas et al. 2003). During the learning process, the long-term memory stores information from the working memory in the form of schemas, which link together various pieces of related information into one single unit (Caskurlu et al. 2020). When these schemas are recalled into the working memory - for example, when a certain action is performed, the information is processed as a single unit (Clarke et al. 2005) and enables the learner to process the action as well as additional information. Moreover, schemas enable the automation of the action as a result of a large practice, which allows free memory capacity for other activities because the schema acts as a control element that directly steers the behavior of the person without the necessity of processing it in the working memory (Caskurlu et al. 2020; van Merriënboer and Sweller 2005). The unavailability of such schemas substantiates the reliance on existing strategies to process information, thereby leading to cognitive overload in the individual involved in the co-creational activity.

The CLT provides a framework for investigations into cognitive processes and instructional design (Paas et al. 2003). Therefore, the CLT aims to avoid cognitive overload in an individual by avoiding unnecessary complexity in assimilating information in the process of acquiring new knowledge (van Merriënboer and Sweller 2005). This cognitive overload may occur more rapidly during the work process on the shop floor, particularly when knowledge generation and documentation take place in the middle of an ongoing working process. Against this background, Paas and van Merriënboer (1994) explain the resulting CL not only in terms of the *task* (here, the production of learning material in the co-creation process) but also in terms of the *physical environment* in which the task must be performed as well as the inherent cognitive capacities of the *employee* itself. *Task* describes the effects on the CL that arise from the co-creation process of learning material and those that are related to the characteristics of the task. A few examples of task factors are visual or auditory overloading caused by the provision of too much information in the task (Mayer and Moreno 2003). The *physical environment* represents a wide range of physical characteristics in which the task and the learning takes place. For example, this includes characteristics such as noise pollution, color, or size of the environment (Vredeveldt et al. 2011). Furthermore, environmental factors describe factors around CL that occur when interacting with this environment. The *employee* describes factors related to the person itself that leads to an increased CL in the interaction with the physical environment and the task. An example of such factors is the IT acceptance of the person (Davis 1989). To ensure that the co-creation process is not perceived as a burdensome additional task and to ensure the acceptance of the system, employees must be made aware of the added value of using it for their daily work. These effects must be taken into account when designing the co-creation process and learning environments in general.

The CLT aims to guide instructional designers to (1) consider differences and cognitive abilities of employees to prevent cognitive overload (Caskurlu et al. 2020), (2) deal with complex tasks (Huh et al. 2019; Janson et al. 2020), and (3) help employees to develop knowledge and skills (Leppink et al. 2013). To achieve these goals, CLT provides instructional design strategies, which are empirically validated, to prevent increased CL in the learning outcomes in different settings, including learning settings (Deng et al. 2020). Various studies provide guidelines to design learning environments to prevent CL. For example, Mayer and Moreno (2003) introduce nine strategies to prevent cognitive load in multimedia learning in general, and Caskurlu et al. (2020) provide guidance for higher education contexts. To date, various studies have shown the effectiveness of such CLT strategies in terms of the learning process and outcomes (Hoffmann et al. 2019; Ayres 2020). Moreover, researchers have also addressed the need for collaboration between practitioners and instructional designers in the design process to manage CL and improve the quality of the co-creation process (Brigance 2011). Therefore, we assume that CL has a negative influence on the ability of employees to co-create learning material, and instructional design strategies can help to prevent cognitive overload in the process. Consequently, we aim to integrate these theoretical aspects into a co-creation system in the domain of the manufacturing industry.

## The Co-Creation System project

**Fig. 1:**
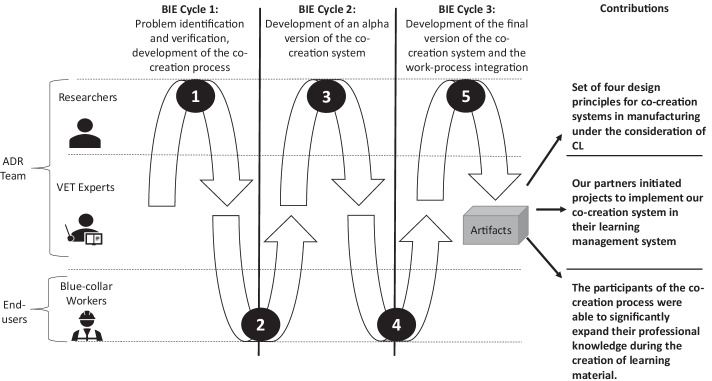
Organization-Dominant BIE of the development of the co-creation system. Adapted from Sein et al. (2011)

In this section, we describe the development process of the design of the co-creation system that supports the cooperation of blue-collar workers on the shop floor. The environment is embedded in a larger ADR project, which aims to design a holistic co-creation system with different modules in order to support learning processes in manufacturing in a sustainable manner (Weinert and Thiel de Gafenco 2020). An overview of the project is presented in Fig. 1 and Table 1.

**Table 1:** BIE process including inputs, used methods, and outputs

BIE Part	Input(s)	Method(s) used	Output(s)
1	Two main problems from the problem formulation stage: • Existing learning material is difficult to use in process-integrated learning. • Lack of didactic knowledge for the development of learning materials.	Work process analysis and observational interviews within two manufacturing companies for two days. Narrative literature review.	Initial set of design features for rapid prototyping approach, practical, and theoretical requirements.
2	Initial set of design features for the co-creation system.	Focus group workshop in manufacturing in a health care company in China with four employees for two days.	Initial set of four design principles.
3	Feedback that particularly focuses on the scope, completeness, and understandability of the alpha version of the co-creation system.	Iterative refinement of the design principles and elements within the ADR team using workshop settings. Discussions between developers and the VET experts to sharpen the design elements.	Beta version of the co-creation system based on the design principles.
4	Beta version of the co-creation system based on the four design principles.	Focus group workshop with 12 workers and 2 trainers in VET training center in Germany for three days: • Concept map intervention to measure the learning outcomes of the users in the co-creation process. • Usability testing of the system. • Structured video analysis to evaluate problems in the co-creation process	Feedback that particularly focuses on usability, helpfulness, and acceptance. Evidence of improved learning outcomes through co-creation intervention.
5	Feedback that particularly focuses on usability, helpfulness, and acceptance.	Iterative refinement of the design principles and elements within the ADR team using workshop settings. Focus group workshop with 18 workers in manufacturing in a health care company over two days: • Concept maps to measure the learning outcomes of the workers. • Questionnaires to measure the cognitive load during the co-creation of learning material.	Final set of design principles. Evidence of low cognitive load of the participants within the co-creation process as well as a demonstration of improvement in employee learning performance. Expansion of the professional knowledge of the employees through the co-creation process.

### Methodological research approach

The considerations above lead to the need for a holistic approach to the design of the co-creation system that can facilitate the employees in the co-creation process of work-process-related learning material. To achieve the research goal and to structure our work, we use the ADR method given by Sein et al. (2011). ADR is a research method that draws on action research and is used to create IT artifacts to solve organizational problems. Action research pursues a practical approach in which hypotheses and their implications lead to a change in the sense of a problem solution (Avison et al. 1999). We follow this approach because we want to (a) solve a specific set of practical problems that practitioners and researchers experience in their work and (b) to contribute to the body of knowledge by designing and evaluating a new artifact (Gregor et al. 2020). In the first stage of the ADR method given by Sein et al. (2011), the emphasis is on problem formulation, whether it is theory-integrated research or a practice-inspired approach. After the formulation of the problem, the BIE cycles are described. Each cycle is followed by a reflection and learning for the redesign of the following cycle. In the following section, we go into more detail regarding the background and the course of our ADR project.

### Stage 1: problem formulation

Over the last years, we have observed a tremendous increase in the number of participants in in-company training in the manufacturing industry and a decrease in the quality of work-process-related learning material (Filipenko et al. 2019; Meinhard and Flake 2018). As already mentioned, this often affects the quality of new employees’ training in problem-solving processes. Since existing material was developed by employees who are not involved in manufacturing and who have no prior didactical knowledge, it is often not suitable as training material. To further validate our experiences, we conducted a work-process analysis (Spöttl 2007) to capture the current work and learning situation of employees in different companies. This input from practitioners and blue-collar workers led to practice-inspired research for developing the co-creation system. In summary, two specific problems were identified. First, existing learning material rarely meets the necessary quality for use in process-integrated learning; second, the creators of learning materials in manufacturing often do not have the necessary prior didactic knowledge to develop work-process-related learning materials.

### Stage 2: organization-dominant building, intervention, and evaluation (BIE)—cycle 1

The second stage of ADR uses the problem formulation of stage one as a platform for generating the initial design of the co-creation system. This development process is sharpened by subsequent design cycles. Figure ‎1 presents the adapted version of the organization-dominant BIE given by Sein et al. (2011). Our ADR team consists of research professionals and VET experts from an inter-company vocational training center that represents the practitioners in this project. In addition, intensive contact and exchange with the company described in Section 2.1 was maintained during the development process of the system. The end users are blue-collar workers in the manufacturing industry - that is, the learners in the learning environment. Three cycles are used for the design of the system. The first BIE cycle consists of the initial requirement development phase, the first alpha version testing of the co-creation system with the practitioners, and the first testing phase with learners in a manufacturing plant of the health care supplier.

#### Development of design elements of the co-creation system for cycle 1

**Fig. 2:**
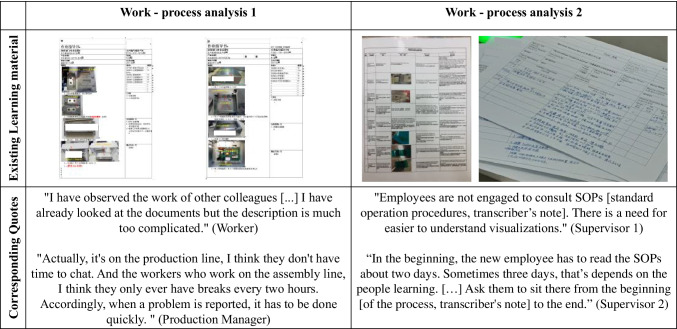
Insights from the interviews and work process analysis from 1) a heating equipment production facility and 2) a cardiac catheter production facility

Addressing the building phase of the first BIE, the following section describes the design elements and is based on the identified requirements from practitioners, end-users, and scientific literature (Appendix 5.). Furthermore, we developed a first version of the instructional design model for the co-creation process based on two work process analyses. Work process analyses are used to analyze existing work processes using structured observations and interviews with employees as well as reviewing existing learning and work tasks to develop work process-oriented learning systems (Spöttl 2007, Appendix 5.). A first insight into the identified deficiencies in the used learning materials, as well as some corresponding quotes from employees, is presented in Fig. 2.

The co-creation concept consists of four building blocks: (1) Providing structure in the creation process, (2) implementing motivational elements, (3) creating emotional proximity to the learning material, thus fostering responsibility and ownership, and (4) offering feedback on learning material for quality assurance. The learning material was created using a systematic process and posted on a learning management platform. In doing so, the fast and early development of a prototype for testing and readjusting the core functions and elements of the co-creation platform is an important goal within the first development cycle. Prototypes are useful to determine and sharpen requirements, receive user feedback, and identify the risks in a project (Davadiga 2017; Urquijo et al. 1993). The implemented core functions and design elements with the corresponding requirements regarding the CL factors are presented in Table 2. We assign the requirements to the respective design element according to their practical (P) and theoretical (T) background. The complete derivation of the requirements can be found in Appendix 5..

**Table 2:** Developed core design features for the co-creation system (P: Practical Requirements, T: Theoretical Requirements)

Cognitive Load Factors	Design Element	Description	Addressed Req.
Environment	Integration of multimedia content	Multimedia formats such as images and videos enable a visual representation of the actual environment and, thus, a direct situational reference of the learning content in the working context.	P1, P2, T3
Task	Structured guiding questions	Structured questions and work instructions support the employees in the creation of learning content, which guides the employee step by step through the creation process.	P1, P2, T1
	Structured UI	The interface for entering new knowledge elements is reduced to the essential input elements.	P2, T2
Learner	Integration of evaluation and comment functions	Integration of evaluation and commenting functions for the created learning content to develop a sense of responsibility and for discussion and feedback for the creator.	P1, T4, T5
	User profile	Integration of a user profile for employees	P4, T4

##### Environment

Numerous approaches attempt to connect the digital world with the real world (Baik 2012). The integration of multimedia content appears to be trivial here, but in practice, it has been shown that the existing learning and documentation material used in the work process is often offered as pure text blocks or images of bad quality (see Fig. 2).

Although images and videos are becoming increasingly common, they are not kept up to date and are disproportionate to the actual work process, which greatly limits their usefulness for employees. Simultaneously, the use of images to explain texts leads to a reduced CL, with simultaneous improvement in the active processing of information during the subsequent learning process when using the learning material (Schwamborn et al. 2011). With this in mind, we have integrated the possibility of *adding multimedia content*, such as images and videos, to provide employees with a visual representation of the work process, while bearing in mind the same time requirements from CLT (P1, P2, and T3).

##### Task

To avoid the cognitive overload of learners, it is important to reduce unnecessary distractions in the creation process. Therefore, it is important to design the user interface as clearly as possible and in a structured manner, particularly in production, where there is a high potential for distraction from external factors. Vredeveldt et al. (2011) investigated the effects of visual distractions on the CL. Furthermore, in the interviews, the workers mentioned that they often have limited time to document their knowledge (see Fig. 2). They concluded that irrelevant information must be eliminated to avoid CL in the learning process. We have reduced the complexity of the creation process by specifically asking *structured guiding questions* and giving instructions to the employee (Castro-Alonso and Koning 2020) to avoid possible distractions in the knowledge-generation process (P1, P2, and T1). The *UI is restructured* to focus the attention of the user on the co-creation process (P2 and T2).

##### Learner

To achieve high-quality learning material, the employees must become aware of their responsibility toward the creation of learning material (Hall and Stegila 2003). This sense of responsibility can be improved through the interaction with other employees on the learning platform (Pipek et al. 2008). Furthermore, these interactions can be a great source of creativity, which may enhance the quality of co-created learning material (Kohler et al. 2011). The active use of comments or even the mere display of comments can contribute to the improvement of social presence on online platforms and, thus, also to an improved sense of responsibility of the creators (Andel et al. 2020). Therefore, we implemented a *comment and evaluation function* in the co-creation system to enable a discussion regarding the material as well as for feedback for the learner (P1, T4, T5). van Gerven et al. (2002) and Kalyuga et al. (2012) have discovered that current knowledge has a major influence on CL. This estimation was confirmed during the focus group workshop, as more experienced employees were able to handle the prototype much more easily and effectively than employees who did not have a similar experience (for further information, please see Appendix 5.). Furthermore, the *integration of a user profile* in which the experience of the users can be updated is considered an important addition to the co-creation system. Based on this information, the system can adapt the creation process by giving more or less assistance and link the creation of content to a certain experience level of the employees (P4 and T4).

**Fig. 3:**
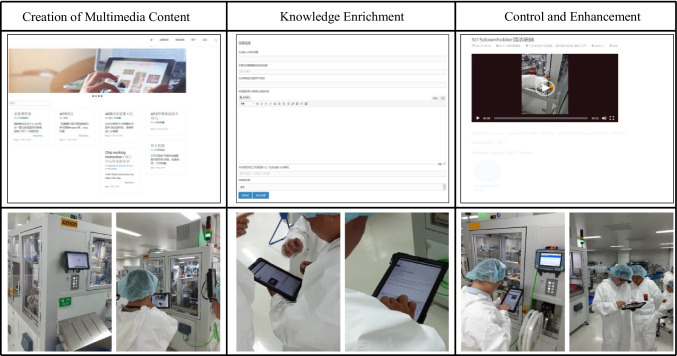
Overview of the Evaluation Workshop (Weinert and Thiel de Gafenco 2020)

In the next step, we developed the first prototype based on the requirements and design features to enable a continuous evaluation, as suggested by the ADR method. The prototype was developed based on the existing content management system (CMS) WordPress using plugins like Bodypress^1^ for organizing the memberships within the CMS and adding a front-end login interface as well as Frontier Post^2^ for adding a post-revision function for the front-end. These plugins were further developed by using PHP and Javascript. The web-based input of the text and multimedia elements was developed using fabricJS^3^. Furthermore, to test the user’s input, it is merged using the TinyMCE editor^4^. For presenting the structure creation process, we used HTML and CSS.^5^. To implement the outlined requirements from above, we developed a first draft of the structured guidelines and UI for the creation of the learning materials (see middle column, Fig. 3). By asking clear questions and avoiding unnecessary functions, attention must be focused on the co-creation process. Moreover, we integrated multimedia features to develop images and videos to further supplement the learning material (take a look at the left column, Fig. 3). To promote the exchange between the supervisor and employees, a review of the learning content takes place after the learning material has been submitted. Simultaneously, colleagues can give hints for the improvement of the material through a comment function (right column, Fig. 3). As a part of this stage, we evaluate the co-creation concept with the learners in their working process in a workshop.

The learning material is published on the co-creation system in the form of a contribution. In the creation process, the learners were required to give a meaningful title, formulate a suitable learning goal for the contribution, indicate the associated work process, and specify the subjectively perceived difficulty (easy, medium, difficult) of executing the documented work process for the created content. Furthermore, the system enables the possibility to add text and multimedia content (images and videos) to the material.

##### Workshop procedure and participants

Three blue-collar workers employed in the plant of the same health care supplier used a prototype in their working process to create work-process-related learning material. The three employees are primarily employed in an automated manufacturing line for the production of catheters. The workers were given the task of developing as many different learning contents as possible within two days. This period was selected because employees could not create learning material during full working hours but only after problem or maintenance work when they only had to observe the running process. At the beginning of the workshop, the employees received a short introduction to the co-creation platform in advance. Furthermore, we used an example to show participants how the process of creating learning materials works. In addition, the participants were given the task of documenting their work processes using a tablet and the co-creation system. In the beginning, the participants recorded rather simple work steps (e.g., refilling storage containers). In the end, they documented increasingly difficult work steps (e.g., when a machine error made further production impossible). During these two days, the developed learning materials were continuously reviewed by the supervisor at the end of the day and then approved or sent back to the employees for revision. Most of the time, the employees performed these revisions when the machine did not indicate any errors and the employees only had to monitor them. During the creation of the learning material, as well as during the revision of the learning material, we observed the participants in order to identify possible weaknesses in the creation process. At the end of each day, an interview was conducted with the staff to ascertain what problems were encountered in the use of the platform and the development of the learning materials.

##### Method

In order to understand further requirements as well as to validate the already collected and implemented ones, we conducted a focus group workshop directly in the work process (Onwuegbuzie et al. 2009). We used observation methods and action-oriented interviews to evaluate our first prototype. With the help of observation methods, the behavior and interaction of the employees during the creation of the learning content can be examined more closely (Becker 2008). At the same time, observation interviews - as a special subtype of observation procedures - offer the possibility to deal more intensively with special work processes (Becker 2008). During the observation interviews, work processes are observed in a structured manner, and based on this, interviews were conducted with the workers in relation to their work activities. These kinds of interviews are often used in work psychology and aim to record the psychological regulation requirements of the work activity that workers engage in (Becker 2008). At the same time, the interviews were intended to identify the potential for improvement and feedback from the learners about the usefulness and usability of the co-creation system. Furthermore, in order to be able to evaluate the quality of the created learning material, the VET experts from the ADR team reviewed the comprehensibility and completeness of the material at the end of the workshops. The interviews and the results of the observation were then evaluated using qualitative content analysis (Mayring 2014) and are presented in Section 3.3.2.

#### Reflection and learning from BIE cycle 1

Based on the results, we were able to derive five recommendations for the further development of our co-creation system. The recommendations and their explanation can be found below. Furthermore, we developed design principles for the co-creation system. These principles will support us in the further development of the platform and will be reviewed at the end of the cycle.

Useful design features and recommendations by the learners:

1. Video functions were often used, but in almost all cases the videos lacked an explanation of the individual work steps.
2. Integrate a drawing environment to highlight certain areas in the photos.
3. Do not present learning objectives to employees. Most work processes are too complex and multilayered, so the employees cannot formulate a learning goal level or a concrete learning objective.
4. Integrate a possibility to choose between a standard and problem-solving process to define the type of working process.
5. Self-assessment of the difficulty is not considered promising, since experienced colleagues consider work steps to be too easy even if they are highly complex.

During the first pilot, we found that many employees used the video function (1). When questioned about this, they told us that occasionally it would be easier to simply make a short video than a picture or text. However, it was found that the independent creation of a short video for the employees was associated with certain difficulties.

“I was afraid of making mistakes and then having to shoot everything again.” (Worker A).

Especially for activities that could not be carried out alone, another colleague always had to be consulted for the video creation. In many cases, this was not possible, which reduced the quality of the resulting learning material. Furthermore, we observed that in cases of creating learning videos, almost no further explanation in the form of text or pictures was provided. This reduced the comprehensibility of the content. Simultaneously, the sound recordings were usually very poor due to the noise level during production, which made the explanations partially incomprehensible. The background noise of the work environment brought additional disturbance to the learners. Similar problems have also been documented in the literature (Vredeveldt et al. 2011); thus, we decided to disable the video function and focus on the photo function. In addition, in our case, we found that employees had an easier time creating photos than they did developing videos. This contributed to lowering the inhibition barrier for the development of learning material.

The second recommendation was requested by many workers who took pictures of their work. “Would it be possible that I could also emphasize something in the picture?” (Worker B). Against this background, the next version of the co-creation system may offer the possibility to highlight certain areas on the images in the form of circles or arrows (2). This feature also reduced the cognitive burden on later learners, simultaneously improved reflection with the created learning material (Koć-Januchta et al. 2020), and also provided a structured guideline for the learners.

During creation, employees must formulate a learning goal for the created learning content. However, we identified that the employees found it very difficult to formulate a concrete learning goal. The reasons for this can be complex: it may be a consequence of a lack of didactic knowledge or insufficient instruction received beforehand. At the same time, however, these difficulties could also be the result of the type of generated micro-learnings. Against this background, the request for learning objectives will be removed (3) in the next version of the co-creation system; instead, the possibility of specifying a standard and a problem case will be offered (4). During the self-assessment of the difficulty of the learning material, we could not detect any problems and the participants in the workshop did not note any problems either. However, when we checked the created learning material, we found that the subjectively perceived difficulty was strongly dependent on the previous knowledge of the individual who created them. This fact is not surprising in retrospect, since other authors have confirmed this connection in several contexts (Hwang and Chang 2011). Against this background, we exclude this function in the second development cycle (5).

##### Design principles

**Fig. 4:**
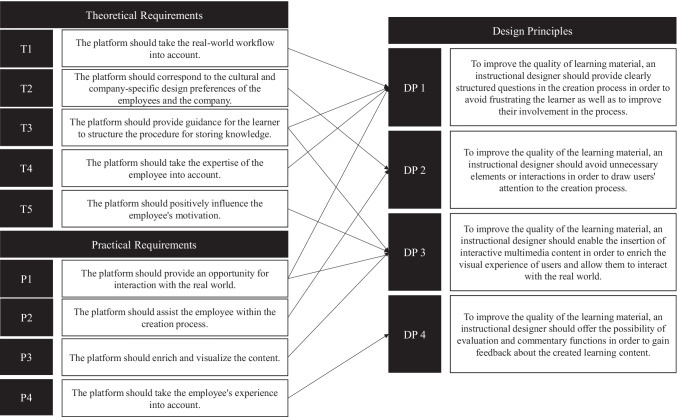
Final design requirements and principles for the co-creation system in manufacturing

Based on these findings, we continue our research by identifying design principles (DPs) for the co-creation system. In the first step, we identify previous design knowledge from the literature (i.e., Morana et al. (2019)), which helps us to specify our design requirements (see Appendix 5.). Second, we use the work by Gregor et al. (2020) to formulate the DPs. For this, we use the components of the design principle schema. An overview of the DPs is provided in Fig. 4.

The employees are not pedagogical experts. They have no experience in designing learning material and do not know which aspects are important for subsequent understanding. Thus, the system can support employees by providing a clear structure of how the learning material must be produced. This was also reflected in the statements of the employees, who showed great uncertainty in the development of the learning materials. Clear questions and work instructions can stimulate the cognitive processes of an employee and make them reflect on their actions (Oeste-Reiss et al. 2016). This has positive effects for the learner - for example, increasing expertise (Damon 1984). Therefore, the co-creation system must provide a structured creation process to support learners during the process. Hence, we make the following suggestion:

- DP1: To increase the quality of learning material, an instructional designer should provide clearly structured questions in the creation process in order to avoid frustrating the learner as well as to improve their involvement in the process.

The creation process of learning material takes place directly within the work process. In this background, employees are concentrating on their work and are confronted with a variety of different influencing factors that can disrupt creative development. Therefore, unnecessary distractions must be avoided in the creation process in order not to unnecessarily burden the employee’s cognitive capacities (Caskurlu et al. 2020). Hence, we make the following suggestion:

- DP2: To increase the quality of the learning material, an instructional designer should avoid unnecessary elements or interactions in order to draw learners’ attention to the creation process.

Reflecting on the created learning material in the workshop for cycle 1, the simple insertion of images and text is not considered sufficient to achieve the necessary reflection of the learning material and simultaneously indicate important aspects in the media material. Therefore, we propose to offer interaction possibilities with which the learner can directly refer to certain content and areas within the pictures. Hence, we make the following suggestion:

- DP3: To increase the quality of the learning material, an instructional designer should enable the insertion of interactive multimedia content in order to enrich the visual experience of learners and allow them to interact with the real world.

A deep understanding of the work process is a necessary precondition for the creation of learning material (Hall and Stegila 2003). This relatively obvious fact is particularly important in the context of the creation of learning material by employees. Different levels of competencies lead to a different understanding of the work process. An employee who can execute a work task but does not know the background of their work finds it more difficult to develop learning material (Hall and Stegila 2003). Simultaneously, it does not appear to make sense to restrict employees in the creation process of learning material based on their previous knowledge, as this may lead to a loss of motivation and, thus, frustration among employees. Therefore, feedback can help to increase the quality of learning material (Ngoon et al. 2018). The feedback can be provided by elaborated elements, which enable exchange among the different employees that are part of the co-creation system. Through this exchange with other employees, the learner can improve their skills in creating learning material (Armisen et al. 2016). Hence, we make the following suggestion:

- DP4: To increase the quality of the learning material, an instructional designer should offer the possibility of evaluation and commentary functions in order to gain feedback about the created learning content.

### Problem (Re)definition

The unanticipated results of the first BIE cycle prompted us to examine more closely a few of the implemented design elements. In particular, the results pertaining to the application of different multimedia contents have caused surprise in our team and also do not appear to have been sufficiently considered in the literature. This appears unusual to us, as many employees considered a video function to be a useful addition during the development of requirements. Unfortunately, we do not have sufficient data at this point to be able to make a definitive assessment. However, we suspect that blue-collar workers underestimated the effort required to develop learning videos in the working process.

After several discussions within the ADR team as well as with managers and responsible persons in the company, we realized that for the mentioned use case - the use of the co-creation system within a partially automated manufacturing process - the use of videos was not promising. The post-processing of the videos is very costly for the people involved (compared to the exchange of texts and images) and the ambient noise in the production environment greatly limits the possibilities of sound recordings. In addition, the use of video recordings appears to demotivate the employees to also textually capture their actions. However, these text-based documentations are very important for the company, as they serve as a basis for optimizing process flows. In addition, it became apparent in our case that the degrees of freedom assumed in the co-creation process (video or photo function, formulation of learning objectives, independent combination of multimedia content with text blocks) overwhelmed the employees rather than supporting their creativity and independence. This is also reflected in the statements of the employees, who expressed concerns about the shooting of the videos and did not know exactly what criteria must be used to develop a good learning video (see Section 3.3.2). At the same time, the functions of the co-creation system must be made obvious so that employees can understand the functions and their effects more easily and quickly (Dix 2007). In order to address these problems and provide better targeted support to employees in the development of learning materials, we initiated a second BIE cycle.

### Stage 3: BIE - cycle 2

#### Development of design elements of the co-creation system for cycle 2

**Fig. 5:**
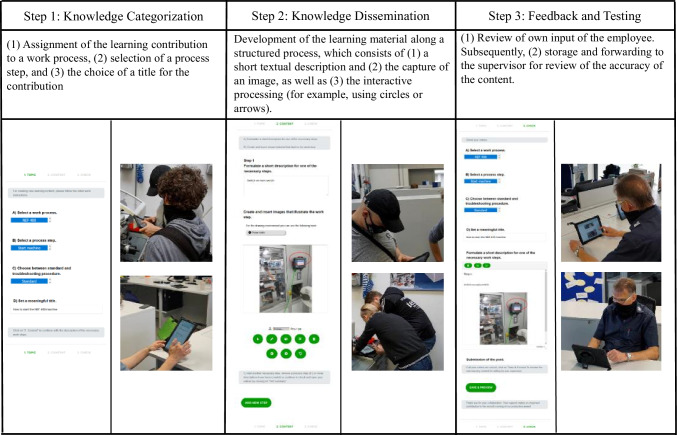
Overview of the designed co-creation system

Informed by the results from the first prototyping and considering the developed design principles and recommendations from the first cycle, we developed a beta version of our co-creation system in cycle 2. To instantiate our tentative design principles, we developed a learning environment in which the co-creation system is integrated. Furthermore, the development of the co-creation system was accompanied by an intensive exchange between the researchers and the practitioners in the ADR team. The developed beta version of the system is illustrated in Fig. 5.

The creation process of learning material consists of three main steps. *In the first step*, the material is assigned to a work and process step. Since the created learning material is situational and consists of rather small-step contributions (Wegener and Leimeister 2012b), as it can be created easily and quickly during the work process, it must be integrated into the corresponding work and process steps. Simultaneously, employees can choose whether they want to document a standard or a problem-solving procedure. *In the second step*, the content of the contribution is created. Descriptions and images are created following a structured learning process and work task consisting of several sub-steps. The task and its individual steps are phrased using directive words, discrete work process identifiers are employed, and otherwise guidelines for Easy-to-Read materials are employed (e.g., Maaß 2015). For every step in a work process, a text and an image are created. The image can be further processed through interaction possibilities. *In the last and third steps*, the employees then check the accuracy of their contributions. Once the learning material has been successfully created, it is forwarded to the responsible supervisor for review. The superior then can revise the article or send it back to the employee with comments for revision, to comment on it directly, and to publish it for the employees afterward. To verify our design considerations, we conducted a focus group workshop. The background and process of the workshop are described below.

##### Method

Evaluation studies indicate that a combination of quantitative and qualitative approaches has proven to be advantageous for obtaining constructive feedback and improving the quality of the co-creation system (Pletz and Zinn 2020). While we capture the aspects of usability and motivation in a standardized manner through questionnaires, factors like learning outcomes are measured with different methods, which are characterized by subjective criteria (Bucher et al. 2019). Furthermore, particularly in manufacturing, there are complex activities involved in the operation of the machines, which contain both declarative and procedural elements - that is, the understanding of the overarching manufacturing concept or the knowledge of the manufacturing steps leading up to the finished product. Therefore, to address this complex evaluation environment (Derry et al. 2010), we combine a structured video analysis and concept maps as our qualitative method approach with the quantitative data obtained from a questionnaire. The video data provide a structured method to make the executed action sequences accessible in their complexity and to enable a comparison with the created learning material. In this manner, we can view the co-creation process from different perspectives, which allows us a more in-depth evaluation of the results (Derry et al. 2010). Furthermore, concept maps are “diagrams that represent ideas as node-link assemblies” (Nesbit and Adesope 2006, p. 413) and are a proven tool for the measurement of learning outcomes (Kinchin et al. 2019). To measure these outcomes as well as previous knowledge, the learners create a concept map at the beginning of the workshop and revise them at the end of the workshop (Liu and Lee 2013). In addition, quantitative questionnaires were used to evaluate the learner experience (Laugwitz et al. 2008), the technology acceptance (Davis 1989), and the motivation of the learners (Kopp et al. 2006).

##### Workshop procedure and participants

**Fig. 6:**
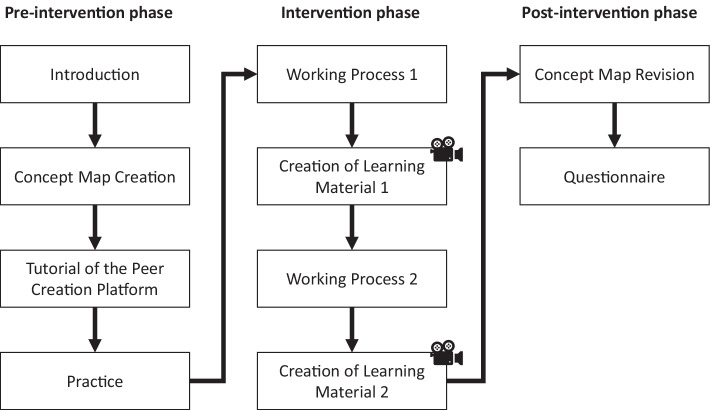
Workshop Design (the sections with the camera symbol are those that were recorded)

The focus group workshop was conducted with 16 employees at the VET Training Center. The workshop situation was selected to meet the conditions in production. The employees here were working on a semi-automated CNC machine, in which certain parts were to be changed and produced anew. At the beginning of the workshop, the employees created an initial concept map to gauge their prior knowledge. After a short introduction to the learning environment, the employees were asked to independently develop learning material in groups of two for a previously defined work process on a partially automated CNC machine. Several types of learning content was created, and the participants alternated in handling the tablet and entries on the learning platform. For the creation, the employees used a tablet to create photo material in addition to their text-based input. During the process, the learners were filmed by several video cameras and observation protocols were recorded in writing. After the creation process was completed, the employees revised their previously designed concept map. In the last step, the participants filled out the questionnaire. The entire procedure is depicted in Fig. 6.

#### Reflection and learning from BIE cycle 2

In this section, we report on the results of the focus group workshop and our conclusions for the further development of our learning environment. To structure our findings, we follow the procedure of the workshop. Accordingly, we first report on the results of the concept maps (both pre- and post-intervention), then show the results of the structured video analysis, and finally discuss the results of the survey.

##### Concept maps

The concept maps were qualitatively evaluated with a holistic scoring rubric (Besterfield-Sacre et al. 2004) that is less likely to award disproportionate scores to long hierarchical structures and, thus favoring the so-called chain maps. Kinchin et al. (2019) do not promote cross-links between hierarchies to boost scores (Novak and Gowin 1984) and do not automatically equate the addition of propositions as desirable map changes as traditional, component-based scoring methods do (e.g., the “Adapted Rubric for Traditional Concept Map Scoring” by Watson et al. (2016)). Instead, the rubric focuses on three distinct attributes present in an individual map: (1) *Comprehensiveness* as an indicator of the breadth and completeness of the externalized knowledge structures, (2) *organization* to describe the effort put into the systematic organization of the concept map as a whole, and (3) *correctness* to identify the degree to which the concept map conforms to facts, known truth, and logic (Besterfield-Sacre et al. 2004). Each of these attributes can be further distinguished by differentiating three performance levels (1 to 3 with their respective criteria for qualitative coding). Appendix 6. presents the resulting coding manual by Besterfield-Sacre et al. (2004) that was used for coding. A total of 16 concept maps from 8 learners were coded in MAXQDA by two researchers of the ADR team after intercoder agreement was established. The mean scores of all learners are presented in Table 3.

**Table 3:** Mean concept map scores of cycle 2

	Pre-intervention	Post-intervention
Holistic Scoring (Scientist)		
Comprehensiveness	1.4	2.1
Organization	1.4	1.8
Correctness	2	2.4
Sums	4.8	6.3

In all cases, the post-intervention concept maps were awarded a higher score than the pre-intervention maps. Changes made to the concept maps benefit Comprehensiveness Scores more (Δ = 0.7) than Organization and Correctness Scores (both Δ = 0.4). Except for one participant, all students decided to revise instead of redo the concept map, followed by the highest summed up Point-Δ of 3 for this participant with 2 additional points in Comprehensiveness and 1 additional Point in Organization.

##### Video data

The results of the structured video analysis revealed exciting further development potential for the co-creation system. Approximately 195 min of video were viewed by two members of the ADR team and then coded using MAXQDA. The coding was supposed to reveal optimization potentials with regard to design, instruction, and interaction. Only the codes that were mentioned at least twice by different participants were recorded. Moreover, design-related recommendations were always taken into account. A brief overview of the definition of these optimization potentials is provided in Table 4.

**Table 4:** Categories for the systematic video analysis

Category	Definition	Codes	Example
Design-related optimization potential	Errors in the platform environment OR difficulties in running the *process* due to the technical design - for example, the given editing possibilities of images.	33	The image was inserted the wrong way around in the learning environment. *(Group2_Video_24:07-24:22)*
Instruction-related optimization potential	Difficulties or errors during the *creation process* due to unclear or unspecific instructions given by the platform - for example, the person asking the trainer or the peer what to do.	24	Participants take several pictures, as they do not know which one they are required to take. *(Group1_Video_14:08 – 14:59)*
Interaction-related optimization potential	Difficulties or errors during the *creation process* due to incomplete knowledge or skills of the person - for example, when the person has difficulties performing the work step correctly or asks the peer what to do.	11	Participants accidentally first carry out and then document the work process. (*Group2_Video_4:59-05:24)*

In the structural video analysis, seven design-related optimization potentials were identified, which will be considered in the next revision of the platform.

With regard to the instruction-related optimization potentials (N = 6), the instructions must be further sharpened in the co-creation process, for example, by providing more information regarding the correct selection of images. Furthermore, the results revealed that the participants were not clear about the difference among work process, process step, and steps within the material. Against this background, we provide more information to inform users about the background of the division.

Within the interaction-related optimization potentials (N = 3), there are approaches for improvement in the introductory tutorial. Several learners had difficulties in the initial phase of the creation process, which could indicate an insufficient introduction to the co-creation system.

##### Questionnaire - descriptive results

The survey was designed to assess the usability of the system (Laugwitz et al. 2008), technology acceptance (Davis 1989), and user motivation (Kopp et al. 2006) during the creation process. To evaluate the usability of the co-creation system, we assess the pragmatic and hedonic quality of our system, where the pragmatic quality captures the objective usability goals and the hedonic quality measures the user satisfaction of the tool. Thus, the usability of the co-creation system is rated as good in terms of both pragmatic quality (+1.15) and hedonic quality (+1.7) (Laugwitz et al. 2008). Furthermore, in order to assess the technology acceptance of our system, we assessed the perceived usefulness and ease of use as well as the behavioral intention to use (Davis 1989). The usefulness of the co-creation process (median = 5.75, interquartile range (IQR²) = 0.75), and the perceived ease of use of the system (median = 5.13, IQR² = 0.88) were rated as high. Furthermore, the intention for the use of the co-creation system in the future was rated as medium to high (median = 5.5, IQR² = 1.25). Furthermore, the results reveal that the employee experienced a strong motivation in the creation process (median = 6, IQR² = 0.33).

After reflecting on these positive results of the testing phase in the VET training center, we decided to conduct another evaluation to test the practicality in the health care company as well. This testing must verify whether the system can also provide these results in practice.

### Stage 4: BIE – cycle 3

#### Development of desgin elements of the co-creation system for cycle 3

Informed by the results﻿ a﻿nd the method of the second BIE cycle, we used the prototype in the company context to verify if the learning environment is suitable for the real-world context (step 5, Fig. ‎1). The aim of the evaluation was the validation of the results of cycle 2 in the manufacturing department of health care providers. In addition, our aim was to examine the cognitive load of the employees during the use of the co-creation system. However, due to the COVID-19 pandemic, we had to radically adjust our methodological approach. To protect employees, we were denied direct access to production because the production of medical devices was considered highly critical during the pandemic. Thus, we adapted our methodological approach to focus on the development of concept maps to capture knowledge acquisition and questionnaires to measure the cognitive load of the employee in the co-creation process.

##### Workshop procedure and participants

The workshop was conducted with 18 employees in a manufacturing plant of a health care company in Germany. Due to the pandemic, travel restrictions, and the lack of equipment, the evaluation could not take place in the same facility that we had visited in cycle 1. The learners were industrial mechanics and mechatronics engineers, who were to use the platform to develop work process-related learning material during the two-day testing. All the learners were males, were on average 19.12 years old, and had 1.3 years of work experience in the company. In addition, the employees were deployed in different work processes, which was reflected in the diverse learning contributions. At the beginning of the two days, the learners were given an introduction to the system and the method of the workshop. Due to the remote situation, the employees, for the most part, familiarized themselves independently. The investigators were available for questions and answers via MS teams.

##### Method

The method of the workshop followed the methodology from cycle 2 (see ‎3.5). Against this background, it will only be presented briefly here. To measure the learning outcomes of the employees, we used concept maps in a pre-post design (Besterfield-Sacre et al. 2004). Furthermore, we surveyed the cognitive load (Ayres and Youssef 2008) and the user experience with the user experience questionnaire (UEQ) (Schrepp et al. 2017). Ayres and Youssef (2008) describe the cognitive load along with different factors, which we have adapted for our co-creation process: *Difficulties in Learning* describe factors that refer to obstacles in the creation process - for example, following the creation process or answering the guiding questions. This factor includes intrinsic and extraneous cognitive load. *Effort and concentration in learning* describe factors that refer to concentration and learning problems. According to Ayres and Youssef (2008), this cannot be referred to as the classical germane load, because the mental effort was completely measured by this schema. Last but not least, the factor *demonstration helpfulness* describes factors to rate the support of the creation process. In their study, Ayres and Youssef (2008) also presented a fourth factor called *motivation*. However, we do not include this factor, because the internal consistency of this factor was rather low in their study. The items can be found in Appendix 7. and were measured on a 9-point-Likert scale.

The UEQ measures employee experience with both pragmatic and hedonic qualities (Schrepp et al. 2017). Both qualities are headings, which summarize different aspects of quality. Pragmatic quality focuses on the goal- and task-oriented aspects of the co-creation system. High pragmatic quality enables users to achieve their goals effectively and efficiently (Laugwitz et al. 2008). In comparison, hedonic quality aims to enlighten other quality aspects that are not task-oriented - for example, originality.

#### Reflection and learning from BIE cycle 3

In this section, we reflect on the results of BIE cycle 3 and the focus group workshop conducted within the working process of 18 blue-collar workers. This last cycle must examine whether the system can demonstrate its usefulness and usability in a corporate context. Thus, within the workshop, 18 learning contents were created, which included a wide variety of topics. An example of such learning content regarding the commissioning of a control cabinet and a coolant analysis is presented in Fig. ‎7.

**Fig. 7:**
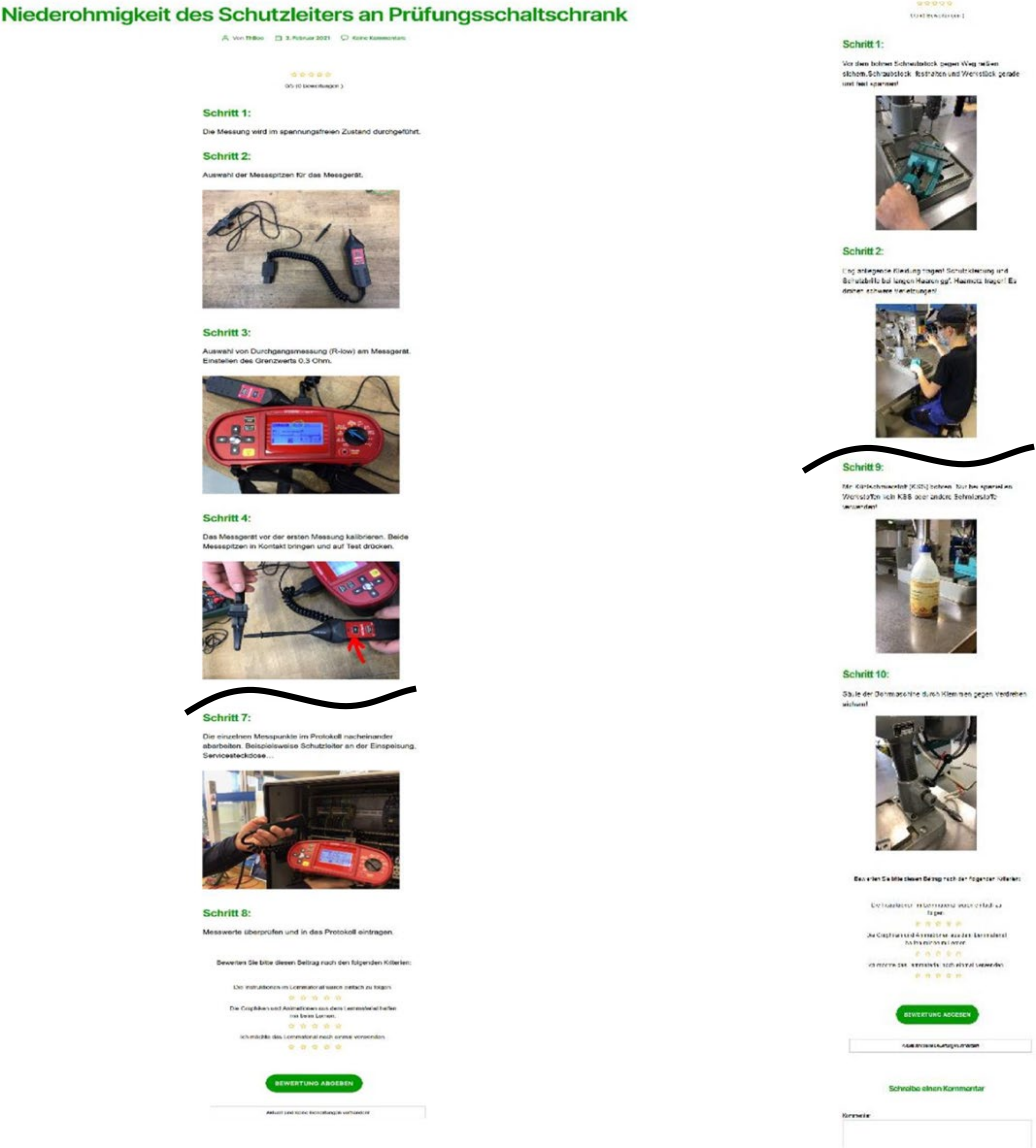
Example of a generated output. Due to the length of the micro-learnings, it had to be shortened

##### Concept maps

As in the procedure outlined in cycle 2, we focus on the three distinct attributes present in an individual map (Besterfield-Sacre et al. 2004, see chapter 3.5.1). In summary, 10 concept maps were analyzed by two independent raters, and several maps had to be excluded because learners had filled them out incorrectly. The mean scores are presented in Table ‎5.

**Table 5:** Mean concept map scores of cycle 3

	Pre-intervention	Post-intervention
Holistic Scoring (Scientist)		
Comprehensiveness	1.9	2.3
Organization	1.7	1.7
Correctness	1.8	2.4
Sums	5.4	6.3

With one exception, learners performed better in the post-intervention concept maps, at least in terms of Comprehensiveness (Δ = 0.4) and Correctness Scores (Δ = 0.6). Organization scores show little change, with one learner’s score decreasing in concept mapping performance in this regard, as the concept hierarchies developed in his map were less distinct. Learners of cycle 3 show a higher level of performance compared to the learners of cycle 2 in both Comprehensiveness and Organization during the first concept mapping phase, but they caught up in Correctness after the intervention.

##### Questionnaire - descriptive results

To measure the usability of the learning environment, we used the UEQ at the end of both days. At the end of the first day, both pragmatic (+2.075) and hedonic (+1.2) qualities had good ratings, which indicates that the familiarization with the system and initial creation of learning materials was easy for employees. On the second day, the pragmatic (1.069) and hedonic qualities (+0.903) were lower, but within the limits specified by Laugwitz et al. (2008) and (Schrepp et al. 2017) for positive usability.

The cognitive load was measured in a pre-post design along with the three factors depicted (Ayres and Youssef 2008). The results of the measurement are presented in Table ‎6.

**Table 6:** Cognitive load measurement

	Minimum	Maximum	Mean	SD
Difficulties in learning	1.9	2.3	2.64	1.68
Effort and concentration in learning	1.7	1.7	5.32	1.30
Demonstration of helpfulness	1.8	2.4	5.94	1.52

The measurement indicates that the cognitive load of the learners was rather low during the creation process. In particular, the “difficulties in learning” factor (which refers to the classic intrinsic and extraneous cognitive load) is rather low, thereby indicating that the co-design process of the learning material is not overwhelming for the workers. Simultaneously, the factor “Effort and concentration during the task” shows an increased value. It can be assumed that the task has been designed in a challenging manner and that the learners do not digress as a result. To sum up, the third factor - “Demonstration of helpfulness” -measures if the co-creation system is considered helpful and does not overburden with extraneous CL. Our results reveal that employees find the learning environment helpful and that it supports them in developing learning materials.

## Discussion and outlook

Overall, the design principles and recommendations presented in this study incorporate specifications for a successful and learner-centered implementation of a co-creation system in the manufacturing industry with the consideration of CL. Through rigorous and formative evaluation within the individual development cycles, we ensured that the identified challenges and problems were addressed. To answer our research question, we provided empirically validated design principles and features for the design of a co-creation system, which fosters the collaboration of blue-collar workers on the shop floor.

### Discussion of the findings

The quantitative descriptive results revealed positive technology acceptance in terms of the perceived usefulness, ease of use, and behavioral intention to use by the employees. This indicates that the learning environment has been designed in such a manner that it can support employees in the development of work-process-related learning materials. The positive technology acceptance of the employees is the central building block for the integration of the co-creation system into their everyday work (Egger-Lampl et al. 2019). Simultaneously, our positive usability tests in cycles 2 and 3 indicate that the learning environment is easy for workers to use, thereby suggesting that workers will use the co-creation system to support the creation process. This assumption is also supported by the positive results in the survey pertaining to user motivation. In addition, during the third BIE cycle, we were able to show that the cognitive load on employees during the creation process was at a manageable level. The co-creation process developed during the design cycles does not appear to overwhelm employees in the creation of work process-related learning materials. Furthermore, the selected measurement method, which we adapted from Ayres and Youssef (2008), does not fit the exact understanding of cognitive load presented by Choi et al. (2014) at the beginning of our study. In most cases, cognitive load is measured according to a traditional understanding. Nevertheless, we chose this measurement method because the evaluation of the instructional design by Ayres and Youssef (2008) and that in our paper are rather similar.

Moreover, the results from the concept map reveal an overall improvement in mapping performance, thereby indicating noteworthy knowledge acquisition achieved by participating in the co-creation process. The individual concept maps indicate a tendency toward prototypical chain maps (Kinchin et al. 2000) that are associated with practical but not expert knowledge regarding the work process (Kinchin et al. 2019). In particular, when pre- and post-intervention maps of individual learners are compared, new propositions are often simply added to the lower levels of the hierarchy, which may be a result of our offer to revise instead of redo the concept map in the post-intervention phase, eventually stunting the reorganization of the map as a whole. Against the background of improving our collaborative training-on-the-job program (Besterfield-Sacre et al. 2004), promoting further learning endeavors to build upon existing knowledge structures (Ausubel 1968) demands both breadth of knowledge and reorganization to be advocated in the co-creation process.

These results reveal that the designed co-creation process can systematically support employees in the creation of their work-process-related learning material. Simultaneously, our results also reveal that the process not only enables the design of didactically high-quality learning material but also leads to positive effects in the knowledge acquisition of the people involved in the knowledge-creation process. Furthermore, the results regarding the motivation of the users also indicate that the design of the co-creation process can increase employees’ willingness to share their knowledge.

Furthermore, various design-, instruction-, and interaction-related optimization potentials for the co-creation system could be identified due to the systematic video analysis, the feedback of the workers, and the insights from the questionnaires. Furthermore, the evaluation directly in the work process in cycle 1 provided additional important insights for the further development of the learning environment. Our approach has revealed how important the structured co-creation process is for the development of high-quality learning material in manufacturing. In contrast to other fields of application where co-creation processes can be designed more freely (Bovill 2020), the use of structured work instructions appears to be promising in such learning-hostile manufacturing environments. This became apparent when we closely examined the individual cycles of our project. During the second BIE cycle, the learning environment allowed a lot of freedom (texts, photos, and videos could be freely placed in the article). Although this allowed the employees to work more creatively, these degrees of freedom in the creation process were more of a distraction during the work process than a positive influence on the quality of the learning material. In comparison, we found that the structured co-creation process in the final prototype had a positive effect on employees to create learning material in the work process.

Overall, employees were positive with regard to the practice of collaboratively developing learning materials through the learning environment. Through collaboration and discussion in the creation process, employees were able to gain new insights into the design of their learning materials. Furthermore, the evaluation of the third cycle revealed that employees occasionally encountered difficulties in developing learning materials collaboratively. As the employees were supposed to create the learning material during the work process, they occasionally had problems finding a partner for the creation. This can be remedied by identifying a means to discuss learning materials through the system. In this context, the question arises regarding whether the review of the learning material by the supervisor as part of the review process is the right way to obtain high-quality learning material. Through this review, employees felt compelled to record only officially approved workflows and did not share their informal knowledge with others (Spence and Reddy 2012). This discussion function could perhaps also help to solve this problem by allowing other employees to report on the informal procedure as well. We believe that these could be exciting aspects for further CSCW research.

As noted in the problem formulation stage, the inclusion of different learners in the co-creation process is a crucial factor for their success (Bovill et al. 2016). During our evaluations, we appear to have succeeded in this because during the structured video analysis, we did not notice any situation in which employees did not participate in the co-creation process. However, at the same time, this assessment must be viewed with great caution. Although the experimenters did not constantly stand next to the employees during the creation process so that they could work as independently as possible, and the cameras were mounted on tripods, it is possible that open observation can cause a behavioral change (Döring and Bortz 2016). While we took great care in our evaluation, the evaluation of cycles 2 and 3 were limited to two days each. Against this background, an in-depth evaluation over a longer period directly within the operation of the learning environment to observe its use in regular operation could be useful. This will allow us to draw further conclusions about the use of the system. However, due to the small number of learners and the given framework conditions, a quantitative measurement of the cognitive load was not possible.

### Contribution to theory and practice

Overall, our study provides several insights and contributions to theory and practice. According to Gregor and Hevner (2013) and Hevner et al. (2004), the theoretical contribution comprises the representation of a real-world problem - that is, design principles for co-creating a learning environment - and enables the exploration of the effects of design decisions and the changes in the real world in order to integrate the co-creation system in the working processes of companies. Following Gregor (2006), this is a theory of “design and action”. We contribute to the scientific body of knowledge by identifying means to overcome the challenges identified by insights gained in the several development steps of the co-creation system. Moreover, our solution reveals an improvement for a known class of problems. According to Gregor and Hevner (2013), our generated design knowledge in the form of design principles is a nascent theory of design and action because it provides generalizable insights that can be applied to various co-creation cases - for example, for digital citizen participation. Furthermore, for the CSCW community, we show how co-creation systems can support the collaboration of employees in designing work-process-related learning material to support the on-the-job training of employees (Ludwig et al. 2018). Simultaneously, we show that such an approach can promote the development of integrative and comprehensive knowledge in the manufacturing industry.

In addition, through our mixed-methods approach, we demonstrate an innovative and interdisciplinary method to make the success of the developed artifacts measurable in small groups of employees, like in manufacturing. In addition, our results reveal that developing learning materials with peers has a positive effect on the learning process of the workers. For example, it appears that through collaborative work on learning materials and the discussions that this stimulated, workers were inspired to create learning materials. As the evaluation for the third cycle revealed, the support provided by the co-creation system in combination with the co-creation of the learning material with a colleague can represent a profitable approach to creating high-quality learning materials in the work process. By combining concept maps, structured video analysis, and descriptive surveys, we were able to measure the effects of our system on the co-creation process as well as identify problems and opportunities for further development of the learning environment.

Finally, we contribute to practice by providing knowledge that supports employees to systematically document their knowledge in the form of work-process-related learning material. This could be a new approach for practice - to make informal knowledge from practice available to other employees in the form of work-process-related learning material (Spence and Reddy 2012). In addition, our research can be considered a starting point for the further development of co-creation systems in other manufacturing areas. By systematically identifying the problems and challenges of co-creation processes in manufacturing, we can support practice in the development of appropriate systems for the creation of work-process-related learning material. To ensure the quality of our design principles and our design knowledge in general, we followed Sein et al.’s (2011) suggestions for good action design research. In addition, we followed the assumptions by Gregor et al. (2020) for the formulation and systematic presentation of design principles.

### Limitations and future research

Although our research of the iteratively integrated insights from theory and practical evidence from the focus group workshops on the problem domain of VET reveals both theoretical rigor and practical relevance, our study has several limitations. While we believe that the focus on the theoretical concepts of co-creation, cognitive load, and situated learning theory is the most suitable approach for building our body of knowledge, a systematic approach for the collection of literature could have been more fruitful. However, since our research adopts a very practical and problem-oriented approach, the rigorous identification of practical requirements was the focus of our work. Moreover, our approach is limited to the knowledge we have gained in the course of our cooperation with the two companies examined here. Although our results have been confirmed by VET experts, it cannot be ruled out that with increasing automation of process flows, requirements of the learning environment, and their integration into the work process may remain unconsidered.

Furthermore, the developed review process of learning contributions may encourage workers to create only official learning materials. From an informal learning perspective, this could be problematic, because informal work processes are often an important aspect of daily work (Spence and Reddy 2012). With this in mind, the creation and review process must be re-examined in terms of whether informal knowledge contributions are also submitted or whether this process makes sharing more difficult than encouraging it.

Finally, additional dimensions of design and evaluation should be considered - such as the inclusion of other stakeholders, more outcome examination, and the consideration of the subsequent use of the created learning material - to ensure that it can improve the learning success of employees. This fact is of profound importance, as manufacturing processes and environments can differ greatly. Thus, approaches from organizational learning can perhaps help to build a bridge to other application contexts and to further explore this topic in an interdisciplinary manner (Senderek 2016). Against this background, the results that are presented are only partially transferable to other manufacturing processes. Consequently, it would be beneficial for future research to conduct a quantitative evaluation of the design principles to further enhance the internal validity of our results and to further prove their effectiveness in various manufacturing contexts. Furthermore, it must be understood that our research can only be the beginning of a holistic perspective of the supporting possibilities that co-creation systems enable in manufacturing. For example, conversational agents like chatbots may offer the possibility of supporting blue-collar workers individually within the co-creation process of learning materials (Wellsandt et al. 2020; Knote et al. 2021). In addition, new technologies such as virtual reality or augmented reality can support employees in the development of learning materials (Hoffmann et al. 2019) and amplify the situational reference of the materials. However, there remains a lack of concrete support for the development of such learning environments, which must go hand-in-hand with new pedagogical concepts in order to support the co-creation of learning materials.

## Appendix 1. General requirements from scientific literature

We derive theoretical requirements from the CLT and instructional design as well as from suggestions from the literature about co-creation processes. In doing so, we structure our findings as well as the other requirements according to the suggestions described by Choi et al. (2014) and try to address every possible theoretical conflict by conducting a narrative literature review. The findings are shown in Table 7.

The first requirement (T1) addresses the problem that the creation process must be interfaced in the workflow. Since different factors (visual and auditory distractions) can occur in work processes, the platform facilitates content creation at times of little or no disturbances (Choi et al. 2014). For example, an employee can create content at times when he or she is only preoccupied with menial monitoring tasks. The second requirement (T2) regarding the environment refers to the design of the co-creation platform itself. It should abide to design conformities present in different markets that reflect the expectations of the employees (Choi et al. 2014; Ernst et al. 2016) as well as the accustomed design preferences of the employees.

The requirement (T3) for the clear structure of the creation process reflects the need for repeatable routines (Paas 1992; Parmigiani and Howard-Grenville 2011) to reduce the cognitive load. As Mayer and Moreno (2003) mentioned, simultaneous addressing of channels (auditory and visual) with different information should be avoided.

**Table 7:** Requirements from theory

Cognitive Load Factors	Derived Requirements from Theory (T)
Environment	T1) The platform should take the real-world workflow into account. T2) The platform should correspond to the cultural and company-specific design preferences of the employees and the company.
Task	T3) The platform should provide guidance for the learner to structure the procedure for storing knowledge.
Learner	T4) The platform should take the expertise of the employee into account. T5) The platform should positively influence the employee’s motivation.

Two requirements regarding the consideration of the learner could be identified. The first requirement (**T4**) relates to the learner’s expertise. The platform should be able to adapt to the level of experience of the employee, especially in the form of additional support during the creation process for less experienced employees (Kalyuga et al. 2012). This notion of experience encompasses the work process knowledge as well as experience in the creation process of learning material. Moreover, elements that improve the motivation of learners to generate and document knowledge in the co-creation learning environment should be implemented on the platform (**T5**) (Putz and Treiblmaier 2015; Schneider et al. 2018). For example, Putz and Treiblmaier (2015) show that the use of gamification elements can have a positive effect on the CL (Schöbel et al. 2020).

### Requirements from practice

We derive practical requirements for our co-creation learning environment, we gathered requirements in two work process analyses and a requirement workshop. In order to gain a better understanding of the current work situation of the employees, as well as to maintain the physical environment of the employees in the production context, a work process analysis was carried out as a first step (Spöttl 2007). Based on these findings, a focus workshop was carried out in November 2019 in order to uncover possible further requirements. Following Greenbaum (2002), focus group workshops are an effective way for learners to generate ideas.

The two work process analyses took place in the shop floor production of two companies in China. The first analysis took place in the production of a heating manufacturer, the second one in a production facility of an international health care supplier. Especially in China, employees depend on in-company trainings, since practical vocational training takes place nearly exclusively there. Thus, process documentation material is often used for the training of employees, which was confirmed by the conducted work process analysis. This documentation material rarely meets the affordances of didactic learning material. As part of the work process analysis, the work processes were reviewed by two members of the research team and one member of the VET expert team. The process includes the analysis of the learning and work process material used as well as interviews with workers and the responsible vocational training personnel in the company.

The focus group workshop was conducted with one experienced technical employee, an operational manager, who is responsible for the supervision of different automated production lines as well as the training operator responsible for the VET in the company. The group was selected to include all stakeholders involved in the co-creation process. The two workers were both involved in production as part of the creation process and were thus able to report directly on their experiences. The manager or supervisor was responsible for reviewing the learning material and was, therefore, the expert on the specific work process of the workers, which was used to develop the learning materials in the workshop. The employees are experienced in the production process and had an average length of service of 1.6 years. This is particularly high for China, which has an average employee turnover rate of over 20 % (Anvari et al. 2014). The operation manager had been working for the company for 8 years.

The overall goal of the workshop was to identify requirements for the co-creation system and the underlying instructional design to reduce CL in the creation process of work-based learning material. A total of four requirements were identified. In doing so, we address the three different causal factors of CL, as described by Choi et al. (2014), with the identified requirements. The requirements are presented in Table 8.

**Table 8:** Requirements from practice

Cognitive Load Factors	Requirements from Practical Application (P)
Environment	P1) The platform should provide an opportunity for interaction with the real world.
Task	P2) The platform should assist the employee within the creation process. P3) The platform should enrich and visualize the content.
Learner	P4) The platform should take the employee’s experience into account.

In digital learning environments, there is often a lack of interactivity, e.g., to enable learners to actively participate in the learning process (Winne 2016). This lack is also mentioned by the learners (**P1**). Especially in training situations, as well as in the creation process, the learners need an opportunity to combine the creation process in co-creation system with the real world.

Regarding the creation task, two requirements were identified. All participants of the workshop mentioned that the creation process is very time-consuming and perceived as costly. Therefore, the platform should support the employees during the creation process (**P2**) to reduce the CL. On the other hand, the employees oftentimes use pictures and videos to enrich their content in the co-creation process because they have difficulties in describing work-related problem cases or the work process itself (**P3**). Therefore, the co-creation system should help the creators to enrich their content with multimedia content. Due to the learning effects that result from the creation of learning material in the co-creation system, the employee needs less support over time, affirming the temporary nature of scaffolding as an instructional method. This effect was confirmed by the learners. Moreover, the level of knowledge among the employees differs naturally. During the workshop, employees with more experience could create content more easily than employees with less work experience. Therefore, the co-creation system should take the employee’s experience into account (**P4**).

## Appendix 2 Results from concept maps

For the qualitative evaluation of the learners’ concept maps, we applied the holistic scoring rubric by Besterfield-Sacre et al. (2004). Table 9 below shows the coding manual used to describe the concept maps’ quality facets Table 10.

**Table 9:** Concept map scoring rubic by Besterfield-Sacre et al. (2004, p. 113)

	1	2	3
*Comprehensiveness –* covering completely/broadly	The map lacks subject definition; the knowledge is very simple and/ or limited. Limited breadth of concepts (i.e., minimal coverage of coursework, little or no mention of employment, and/ or lifelong learning). The map barely covers some of the qualities of the subject area.	The map has adequate subject definition, but knowledge is limited in some areas (i.e., much of the coursework is mentioned but one or two of the main aspects are missing). Map suggests a somewhat narrow understanding of the subject matter.	The map completely defines the subject area. The content lacks no more than one extension area (i.e., most of the relevant extension areas including lifelong learning, employment, people, etc. are mentioned).
*Organization –* to arrange by systematic planning and united effort	The map is arranged with concepts only linearly connected. There are (or no) connections within/ between the branches. Concepts are not well integrated.	The map has adequate organization with some within/ between branch connections. Some, but not complete, integration of branches is apparent. A few feedback loops may exist.	The map is well organized with concept integration and the use of feedback loops. Sophisticated branch structure and connectivity.
*Correctness – conforming to or agreeing with the fact, logic, or known truth*	The map is naïve and contains misconceptions about the subject area; inappropriate words or terms are used. The map documents an inaccurate understanding of certain subject matter.	The map has few subject matter inaccuracies; most links are correct. There may be a few spelling and grammatical errors.	The map integrates concepts properly and reflects an accurate understanding of subject matter meaning little or no misconceptions, spelling/ grammatical errors.

## Appendix 3 Cognitive load measurement

**Table 10:** Items for the Cognitive Load Measurement adapted from Ayres and Youssef (2008)

Construct	Item
Difficulties in learning	How difficult was it for you to follow the instructions in the creation process? (Pre)
	How difficult was it for you to learn from the co-creation process? (Pre)
	How difficult was it for you to understand the characteristics of good learning materials? (Pre)
	How difficult was it for you to internalize the characteristics of good learning materials? (Pre)
	How difficult was it to solve the work instructions? (Post)
Effort and concentration in learning	How much effort did you put into learning the creation process? (Pre)
	What was your concentration level when you were trying to learn the creation process? (Pre)
	How much mental effort did you put into the process of creating the learning material? (Post)
	How much mental effort did you put into solving the work instructions? (Post)
	How much did you focus on internalizing the creation process? (Post)
Demonstration of helpfulness	How helpful were the work instructions for internalizing the creation process? (Post)
	How helpful were the work instructions in understanding the creation process? (Post)

## Footnotes

1
https://de.wordpress.org/plugins/buddypress/.

2
https://de.wordpress.org/plugins/frontier-post/.

3
http://fabricjs.com/.

4
https://www.tiny.cloud/.

5 The source code will be made available via GitHub after acceptance.
